# Magnetite nanoparticles for nonradionuclide brachytherapy^1^

**DOI:** 10.1107/S1600576715008900

**Published:** 2015-05-22

**Authors:** Victor Safronov, Evgeny Sozontov, Mikhail Polikarpov

**Affiliations:** a Research Center ‘Space Materials Science’, Shubnikov Institute of Crystallography, Russian Academy of Sciences, Akademicheskaya 8, Kaluga, 248640, Russian Federation; b National Research Centre ‘Kurchatov Institute’, Akademika Kurchatova 1, Moscow, 123182, Russian Federation

**Keywords:** magnetite, nanoparticles, brachytherapy

## Abstract

Magnetite nanoparticles possess several properties that can make them useful for targeted delivery of radiation to tumors for the purpose of brachytherapy. Such particles are biodegradable and magnetic and can emit secondary radiation when irradiated by an external source. In this work, the dose distribution around a magnetite particle of 10 nm diameter being irradiated by monochromatic X-rays with energies in the range 4–60 keV is calculated.

## Abstract 2

Monte Carlo simulation of the dose distribution in water around magnetite nanoparticles irradiated by monochromatic X-rays is presented.

## 1. Introduction   

Brachytherapy is a radiation therapy method, which is also known as internal radiotherapy. This localized high-technology method is often used for cancer treatment as a complementary technique to conventional surgery, chemotherapy and external beam radiation therapy. Traditionally, brachytherapy involves use of radioactive isotopes that are very problematic in terms of production, handling and disposal. In recent times some attempts have been made to replace this source of radiation with compact low-energy X-ray generating devices and to develop different approaches of radiation delivery to tumors. One such approach is to construct an X-ray tube that is miniature enough to be implanted in or near the tumor as a part of a needle assembly (Rivard *et al.*, 2006 ▶). A different way is to utilize X-ray fluorescent radiation from a secondary target located at the end of a hollow needle and irradiated though the needle opening by an external X-ray source (Liu *et al.*, 2010 ▶). The main advantages of the insertable X-ray generating devices are that they offer a controllable on/off mode, an adjustable dose rate and the ability to function in a conventional operating room with almost no radiological exposure to staff.

Another direction of efforts is to enhance the dose locally by injection of heavy element nanoparticles into a tumor (Hainfeld *et al.*, 2004 ▶). Upon irradiation (with either an implanted brachytherapy source or an external X-ray beam) such particles emit secondary X-ray photons and electrons (photoelectrons and Auger electrons) that cause additional DNA damage in surrounding tissue. Auger electrons produce a stronger destructive effect on DNA than higher-energy photoelectrons (Panyutin & Neumann, 2005 ▶) owing to their more frequent acts of interaction with matter along their path. The spacing between such interactions appears to be comparable to the double helix diameter, which with high probability causes multiple double-strand breaks that are very difficult to repair. However, the penetration depth of Auger electrons is very small, and hence the nanoparticle must be delivered into the cell nucleus or very near to it. The most typical elements used as a source of secondary radiation in such experiments are gold (as metal) and gadolinium (usually as gadolinium chelates). A technique of transportation into the cell nucleus was reported for the case of gold nanoparticles (Dam *et al.*, 2012 ▶). For the case of gadolinium, there exists a very interesting approach of controlled self-assembly of nanoparticles inside a living cell (Cao *et al.*, 2013 ▶).

In this paper we consider another material, magnetite (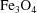), as nanoparticles for local dose enhancement. Magnetite possesses several appropriate features. First, it is biodegradable (Gabbasov *et al.*, 2013 ▶). Second, owing to their magnetic properties such particles can be fixed near the desired location inside the body using external magnets. Finally, magnetite can emit secondary radiation when irradiated by an external source, such as an X-ray tube or synchrotron. We consider two ways to generate secondary radiation: photoelectronic processes and the Mössbauer effect (in the latter case the particles must have a substantial 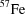 isotope component). In the present work we have simulated dose enhancement around such a particle considering photoelectronic processes only. In future work we will compare the efficiency of such a setup with that based on the Mössbauer effect. The topic of transportation of nanoparticles into a cell nucleus is beyond the scope of the present work.

## 2. Computational details   

The simulations were done using the *Geant4* package (version 10.00.p01; Agostinelli *et al.*, 2003 ▶; Allison *et al.*, 2006 ▶) with low-energy data pack *G4EMLOW*, which includes data from EPDL97 (Evaluated Photon Data Library; Lawrence Livermore National Laboratory, CS, USA, and NIST, Gaithersburg, MD, USA). This library is among those recommended for medical computations by the American Association of Physicists in Medicine (Rivard *et al.*, 2004 ▶).

A spherical magnetite particle of 10 nm diameter was placed at the center of a cube with 100 nm side length. For obtaining dose distributions, the cube was filled with water; for obtaining secondary spectra, it was empty. To achieve good statistics the number of incident photons was chosen to be 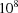 for the case of narrow-beam irradiation and 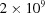 for the wide-beam case (with the exception of the 59.3 keV beam, when we had to use three times more photons because of their weak interaction with matter). Absorbed doses were calculated by accumulating the energy deposited into a spatial histogram with ring-shaped bins of 1 nm height and width, followed by normalization by bin volume. The dose enhancement ratio was calculated by dividing dose values computed with and without the magnetite particle. The step length of continuous processes was limited to 1 nm. The histogram bin size for obtaining secondary spectra was 100 eV.

## 3. Results and discussion   

**Figure 1:**
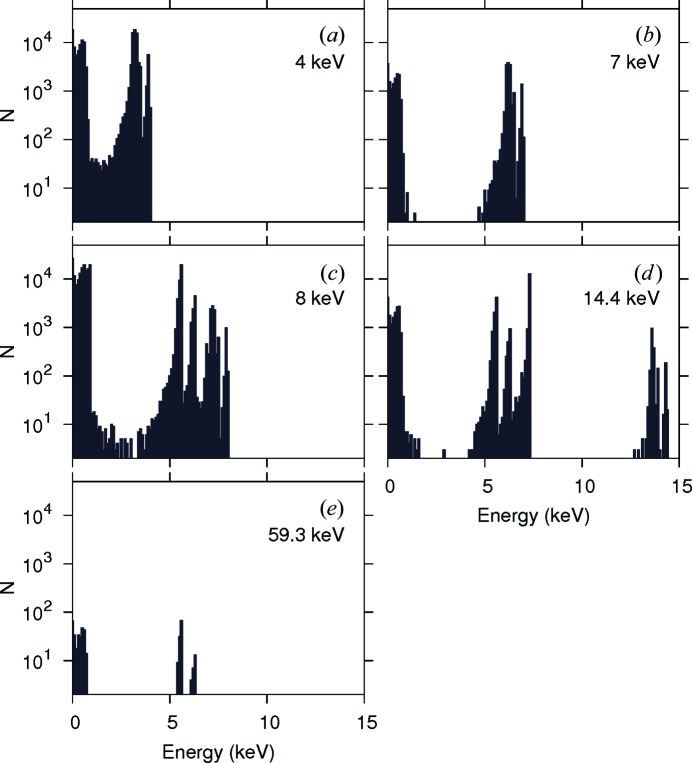
Spectra of electrons emitted by a magnetite particle placed in vacuum and irradiated by a monochromatic beam of photons with the indicated energy. The number of incident photons is 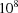 for all plots.

**Figure 2:**
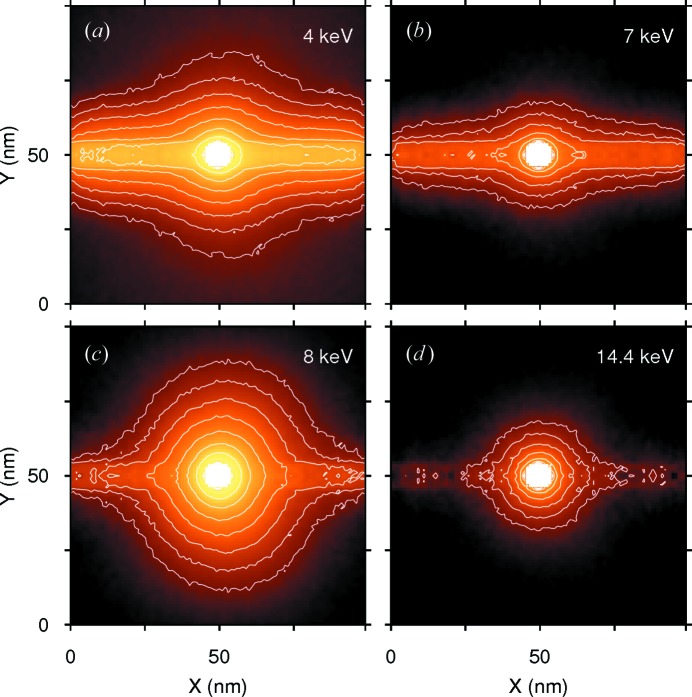
Dose distributions (in arbitrary units) in water around a magnetite particle irradiated by a narrow monochromatic beam of photons with the indicated energy. The beam direction is left to right. Contour levels are the same for all plots. From contour to contour the dose increases twice. The number of incident photons is 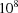 for all plots.

As the first step we made somewhat idealistic computations to clarify the effect of the particle itself. To achieve this we used monochromatic photon beams of the same diameter as the particle (10 nm). This approach allowed us to get rid of bulk water irradiation caused by the wide beam. Secondary electron spectra for irradiation energies of 4, 7, 8, 14.4 and 59.3 keV are shown in Fig. 1 ▶ (for reference, the Auger energy of iron is about 700 eV). Corresponding distributions of the dose absorbed by surrounding water are shown in Fig. 2 ▶ (the dose for 59.3 keV practically vanishes on this scale and is not shown). From these figures one can see that at very low energies (4 keV) the primary beam strongly interacts with water, making negligible the dose enhancement caused by the particle. Therefore, for therapeutic purposes it will be necessary to reduce the nonlocalized irradiation by cutting off the low-energy part of the X-ray tube spectrum. When the incident photon energy becomes noticeably higher than the *K*-edge energy of iron (7.1 keV), strong emission of secondary photons and electrons from the magnetite particle appears, which sharply increases the dose absorbed by the surrounding water. At higher energies the total efficiency reduces owing to the weaker interaction of the beam with the particle material.

**Figure 3:**
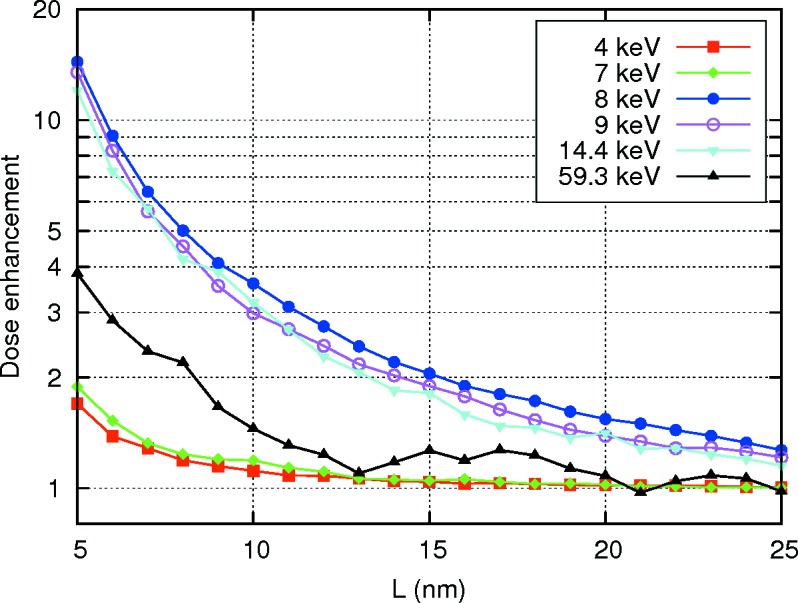
Dose enhancement ratios in water as a function of distance from the particle center in the case of irradiation with a wide beam of photons with different energies.

Then, we calculated dose distributions for the case of wide-beam irradiation, as it would take place in practice. These distributions appeared to be spherically symmetric because of the negligibly small absorption and scattering effects caused by a single particle of such small size and the absence of correlation of secondaries with the primary beam. The dose enhancement ratios against distance from the particle center are plotted in Fig. 3 ▶. From this plot it is clear that the maximal effect is observed at a beam energy of 8 keV. However, even for the case of this optimal energy the region with dose enhancement factor of two or higher appears to be just a 10 nm-thick spherical shell around the particle. Because this is quite a small range, the particles should be somehow transported into the cell nucleus or even into the nucleolus to approach a DNA molecule to damage it. For gold nanoparticles this was reported to be possible (Dam *et al.*, 2012 ▶). We expect it would be also possible for magnetite.

Another problem is the very small penetration depth of 8 keV photons in water (just a few millimetres), which is far from enough for clinical application in most cases. Therefore, the injection of magnetite nanoparticles should be combined with the use of one of the radiation delivery techniques mentioned above (a needle assembly with a miniature X-ray tube or a hollow needle with a secondary target) or used intraoperatively.

## 4. Conclusions   

Magnetite nanoparticles were shown to increase the dose absorbed in surrounding matter upon irradiation. The optimal energy of incident photons is about 8 keV. However, a substantial increase is observed only in the immediate vicinity of the particle. Therefore, to damage cell DNA it would be necessary to transport such particles into the cell nucleus.
